# Environmental colour pattern variation in Mediterranean *Podarcis*

**DOI:** 10.1186/s12862-024-02242-1

**Published:** 2024-04-25

**Authors:** Daniel Escoriza

**Affiliations:** GRECO, University of Girona, Campus de Montilivi, 17071 Girona, Spain

**Keywords:** Competition, Homochromy, Islet, Isolation, Lizard assemblage

## Abstract

**Background:**

Lizards of the genus *Podarcis* are widespread in the Mediterranean region, including islands and island archipelagos. These small-bodied lizards have a predominantly protective green-brown colouration. However, some populations display unusual patterns, in which the colouration is predominantly blue or uniformly black. This study explores the factors that influence this chromatic variation, whether environmental (climate and island conditions) or evolutionary (phylogenetic trait conservatism). The colouration of 1400 individuals (27 species) was analysed in the CIELAB colour space.

**Results:**

Pagel’s λ indicated that colouration is weakly conserved within phylogenetic lineages. Although the island surface plays a key role in the chromatic variability of these lacertids, geographic isolation and climate hold less influence. The colouration of some small island populations tends to be uniform and dark, possibly due to intense intraspecific competition and lower predatory pressure.

**Conclusions:**

This study highlights the importance of island populations in understanding the processes that favour the emergence of extreme phenotypes in small ectothermic vertebrates.

**Supplementary Information:**

The online version contains supplementary material available at 10.1186/s12862-024-02242-1.

## Background

In small vertebrates, body colouration plays a crucial role in individual survival given its protective function [1, 2]. Nevertheless, in some lineages, groups of phylogenetically related species may show significant chromatic variations, which are typically attributable to species-specific habitat preferences [3]. In small ectothermic vertebrates, which are highly exposed to predators, body colouration primarily functions as camouflage and secondarily enables thermoregulation or social signalling [4–6]. However, these roles are not complementary. Dark pigmentation offers superior protection from harmful solar radiation and facilitates thermoregulation via heliothermal conversion [3, 7] but increases individual detectability by avian predators [8]. Consistently, melanic individuals can heat up more quickly than their lighter counterparts but are also exposed to higher predation rates [9].

Lizards of the genus *Podarcis*, which are confined to the warm-tempered zone of Eurasia [10], provide a suitable group of small ectothermic vertebrates to study the trade-off between these opposing roles in colouration. The 27 species of these lizards are present in much of southern and central Europe, as well as in the main Mediterranean archipelagos [11]. They exhibit a broad colour gamut, although most species display variants of green and brown or a combination thereof [10]. This predominance of dull green or brown morphs suggests that the primary function of body colouration in these small lizards is protective [12]. However, some populations have sky-blue or bluish-black colouration, suggesting that there may be some exceptions to this protective role [13]. While these colourations could be due to island syndrome, caused by intense interindividual competition and genetic isolation [13–15], they do not appear in all micro-island populations, even those of the same species.

This study investigates these chromatic variations in *Podarcis* lizards on a large spatial scale. First, we evaluated the possible phylogenetic effect (i.e., the level of trait inertia across the phylogeny) [16]. Then we considered the environmental effect, which can be mediated by climate (e.g., darker colours under cold or rainy conditions or lighter colours in arid zones, i.e., those with substrates devoid of plant cover) [17, 18]. The chromatic patterns could also be affected by island isolation, although isolation does not explain this variation alone, since numerous dull-coloured island populations exist [19–21]. This study tests the following hypothesis: the combined effect of these factors (phylogenetic, climate, island isolation) could explain the chromatic patterns in these small lacertids in Eurasia.

## Results

**Fig. 1:**
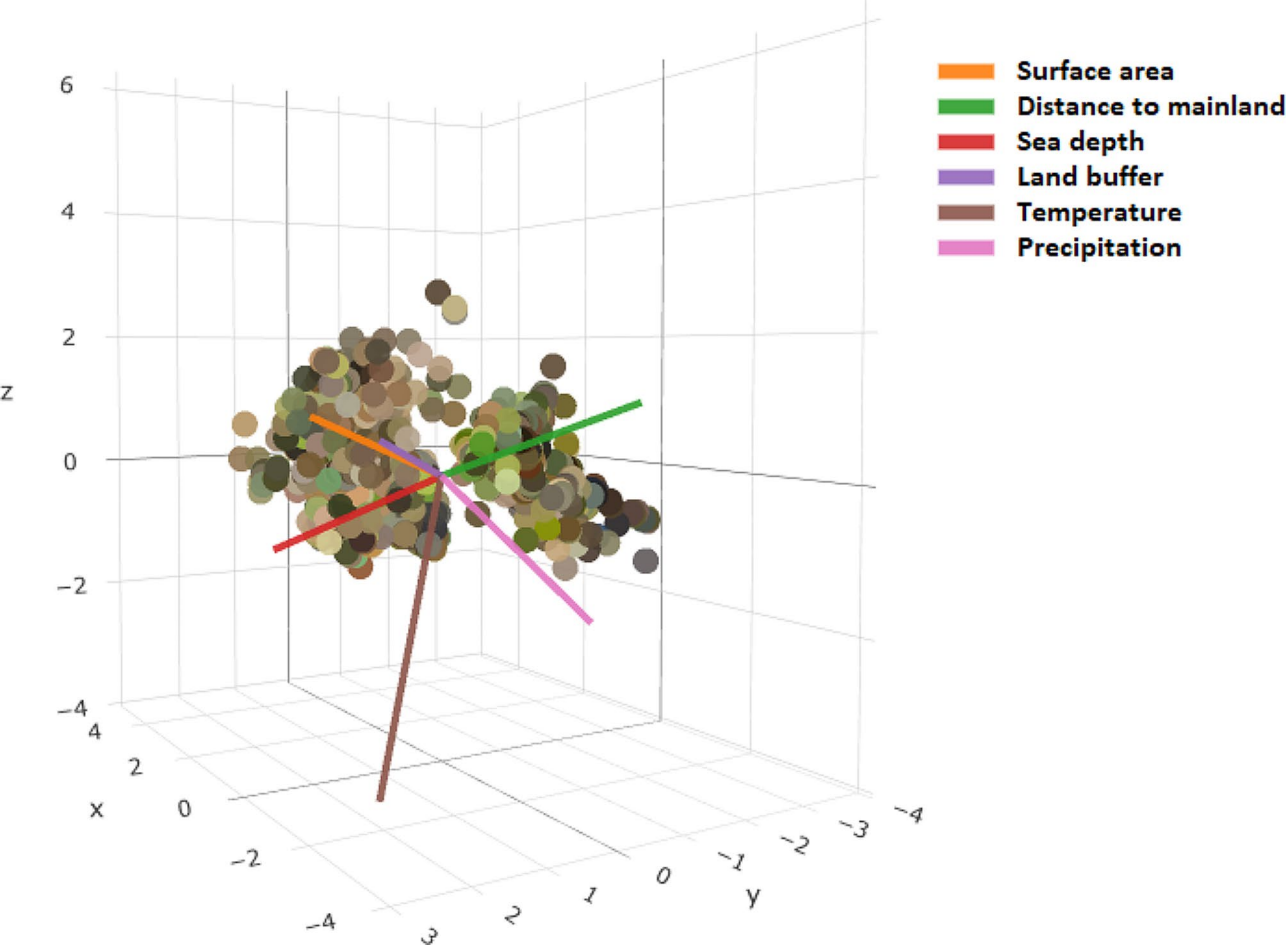
PCA scatter plot showing the association between colouration (average value of CIELAB coordinates per individual) and environmental variables; x-axis proportion of variance = 0.665, y-axis = 0.137, z-axis = 0.081

Estimates of Pagel’s λ indicated a lack of phylogenetic constraints in the chromatic patterns of the lizards (Table 1). Therefore, the phylogenetic effect was considered negligible in the subsequent analyses. The PCA scatter plot (Fig. 1) showed the position of 1400 individual occurrence sites through the environmental space (explained variance = 0.883) (Fig. 1).

**Table 1:** Phylogenetic signal (Pagel’s lambda, λ) in the chromatic variation of lizards of the genus *Podarcis*, estimated after 10,000 resamplings of the response matrix. The mean values of λ ± the standard error are shown. *p*%, proportion of significant *p* values at alpha = 0.05

Variable	λ	*p*%
CIELAB *l*-axis	0.0599 ± 0.002	1.23
CIELAB *a*-axis	0.0824 ± 0.002	1.39
CIELAB *b*-axis	0.0684 ± 0.002	1.32
Distance to centroids	0.1306 ± 0.002	1.58
Colour contrast	0.0904 ± 0.002	1.46

The GLS models built with an exponential and rational quadratic autocorrelation error structure and nugget effect produced the best fit for this data set (Table 2). These models showed that the colour variations were significantly associated with the surface area and the mean annual temperature, although the association with temperature was not maintained after adjusting the *p*-values for multiple testing (Table 3). The association with surface area was positive for the CIELAB *l*-axis (i.e., the individual colouration tended to have lighter pigmentation) and the *b*-axis (i.e., the individual colouration tended to have more yellow pigmentation) and negative with distance to colour centroids (Table 3). These results indicated that the lizards tended to be darker, bluish, uniformly coloured, and atypically coloured with smaller available surface areas (i.e., on small islands).

**Table 2:** Best GLS autoregressive models testing the association between colour variations and environmental variables. The parameters used to build the models with the lowest AICc are shown. AICc, Akaike Information Criterion corrected for finite sample sizes

Dependent	Correlation	Nugget effect	AICc	Delta AICc	AICc weight
CIELAB *l*-axis	Ratio	Yes	11205.4	0.00	0.195
CIELAB *a*-axis	Exponential	Yes	9806.0	0.00	0.163
CIELAB *b*-axis	Ratio	Yes	10456.8	0.00	0.292
Distance to centroids	Exponential	Yes	‒3799.2	0.00	0.957
Colour contrast	Ratio	Yes	9427.1	0.00	0.454

**Table 3:** Best GLS autoregressive models testing the association between colour variations and the environmental variables. The coefficients of the model variables are shown. Significant adjusted *p* values are marked in bold

Dependent	Predictors	Value	t-value	*p*	Adjusted *p*
CIELAB l-axis	Surface area	0.651	4.605	0.00001	**0.0002**
	Distance to mainland	4.613	1.253	0.211	1.000
	Temperature	1.415	0.729	0.466	1.000
CIELAB *a*-axis	Surface area	‒0.314	‒2.643	0.008	0.128
	Average sea depth	0.602	2.472	0.014	0.224
	Temperature	‒4.924	‒1.867	0.062	1.000
	Precipitation	‒1.563	‒1.069	0.285	1.000
CIELAB *b*-axis	Surface area	0.610	4.375	0.00001	**0.0002**
	Average sea depth	‒0.625	‒2.108	0.035	0.560
	Land buffer	1.168	1.819	0.069	1.000
	Temperature	8.196	2.632	0.009	0.144
	Precipitation	2.361	1.526	0.127	1.000
Distance to centroids	Surface area	‒0.004	‒6.901	0.00001	**0.0002**
Colour contrast	Surface area	0.421	4.493	0.00001	**0.0002**
	Distance to mainland	0.111	1.348	0.178	1.000
	Temperature	0.501	0.306	0.760	1.000

## Discussion

This study evaluates the variation in the chromatic patterns of a group of small lacertids that spread throughout the Mediterranean region and occupy diverse continental habitats and islands [22]. Intrageneric colouration patterns lack phylogenetic structure, in contrast to observations in some polytypic species, such as *Podarcis muralis* [23]. The analyses revealed that the development of abnormal chromatic patterns was negatively associated with the insular surface. The results also indicated that the association with these anomalous patterns was independent of climate and island geographic isolation (anomalous patterns appeared on islets close to the continent and islets within archipelagos) [24].

However, the role of climate in part of the chromatic variation was not entirely negligible; the mean temperature showed a weak but recurrent effect in the variability measured in the CIELAB axes. The strongest association identified with climate was with the CIELAB *b-*axis (positive correlation with yellowish colourations), that is, a tendency to light-yellow colours under warmer conditions. The analysis indicated a non-significant association between dark colouration (CIELAB *l-*axis) and cooler temperatures. This finding may be biased due to the presence of melanic populations on the hot, dry Mediterranean islands. These small lacertids with low adult body mass (1.7─2.3 g) [25] can heat rapidly by basking, and therefore the need for energy-efficient colouration may not be as necessary as in larger species of reptiles [26].

Individuals from small islands tend to be darker or bluish and frequently also lack disruptive patterns (they showed more uniform patterns in the analyses). The mainland and larger island populations display shades of green and brown or combinations of the two colours, usually spotted or striped (disruptive pattern) [27]. These patterns are likely protective, matching the colours of the forest floor substrates. This homochromatic colouration reduces the detectability of lizards by daytime predators with colour discrimination systems, such as birds [28]. In other reptiles, such as snakes, disruptive patterns are also typical of species exposed to attack by diurnal birds [29].

Avian predators have an intense impact on lizard populations. Small lacertids can account for up to 50% of prey biomass for common predatory birds, such as shrikes (*Lanius meridionalis*) and kestrels (*Falco tinninculus*) [30, 31]. These predatory birds have excellent tetrachromatic colour vision and the ability to detect colour differences in the blue, brown, green, red, and yellow ranges [32, 33]. Therefore, they respond to variations in the colouration of a lizard, increasing the frequency of attacks against individuals lacking protective patterns [12].

However, small islands occupied by anomalously pigmented lizards typically lack resident populations of land-predator avian species (such as shrikes, kestrels, and similar raptorial birds). These islands do not provide enough prey to maintain breeding populations, or birds must make energetically expensive flights across bays or channels [34]. Raptor species (*F. tinninculus* and *F. eleonorae*) that breed on larger islands show occasional consumption of lacertids, while Yellow-legged Gulls (*Larus michahellis*) avoid them altogether [35]. Raptor predation intensity may also fluctuate depending on the proximity of the islet to larger islands [36]. Therefore, these micro-island populations relatively far from large islands are less or not exposed to predation and can reach extremely high densities (e.g., 1500‒8000 individuals ha^− 1^) [37]. Under these conditions of extreme competition and scarce trophic resources, traits that enhance intraspecific competitive superiority are selected [38].

## Conclusion

The use of web-based community science images allowed specific hypotheses about variations in morphological or life-history traits over broad geographical ranges to be tested. The findings revealed that body colouration in small lizards is not subject to evolutionary constraints and plays a primarily protective function. However, this protective role weakens or even disappears in small islands, where intense competition affects the survival and growth of lizards. These results highlight the importance that lizard island populations have as models for exploring synergistic relationships between phenotype, environment, and social interactions.

## Methods

### Study region and environmental data

**Fig. 2:**
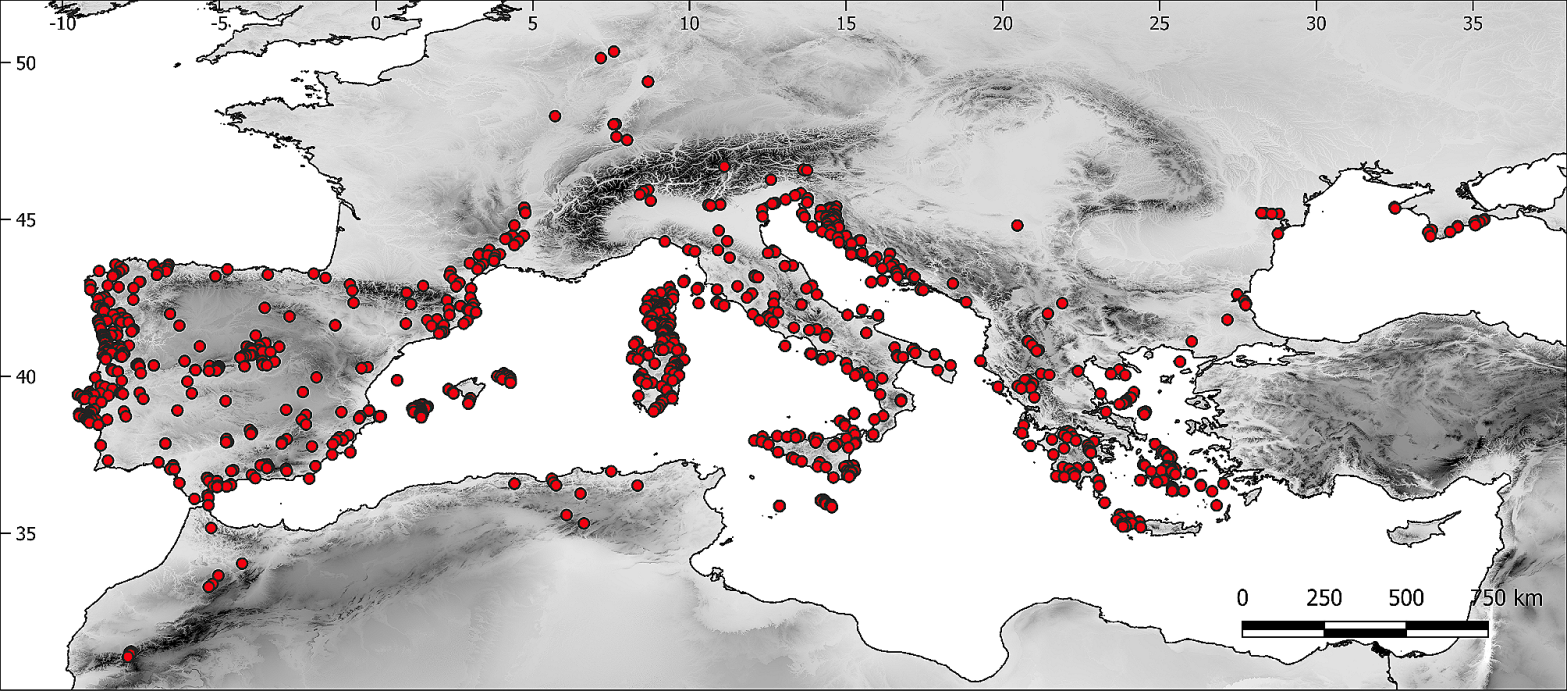
Map of the study region, showing the distribution of the sites of image origin (red circles) with the terrain elevation on a grey-scale background

The study region encompassed the Mediterranean basin, mainland Europe and North-western Africa, approximately between 50º N‒31º N and 9º W‒35º E (Fig. 2), where all species of the genus *Podarcis* are distributed [10]. Regional climates are classified as warm-temperate (mesic Mediterranean and steppe) and cold-temperate (*Csa-Csb*, *BSk*, and *Dfb* Köppen types) [39].

Two ensembles of variables were selected that describe the climatic variation in the region and the size (surface area, in km^2^) and relative isolation of the islands [40]. First, the climatic variables provided the mean temperature in ºC and the accumulated yearly precipitation in mm, as modelled from meteorological data (WorldClim 2 database) [41]. The mean temperature and accumulated precipitation describe the climate properties that determine different thermoregulatory strategies and colouration patterns in lizards [42]. Climate data were obtained with a spatial resolution of 30 arc seconds for each geographic coordinate of the origin of the photographs using the package Quantum-GIS (QGIS Core Team, 2024) [43]. Second, island isolation was evaluated using the shortest distance from the island to the mainland (in km), the average depth of the sea through this distance (in m), and the amount of land area (in km^2^) covered by an offshore island buffer with a radius of 50-km [40]. The average depth of the sea was calculated from a digital model of the sea floor [44], downloaded from the GEBCO database. For localities on the continent, the shortest distance and average sea depth were assigned null values, while the surrounding land area was calculated using the same procedure.

### Individual data

**Fig. 3:**
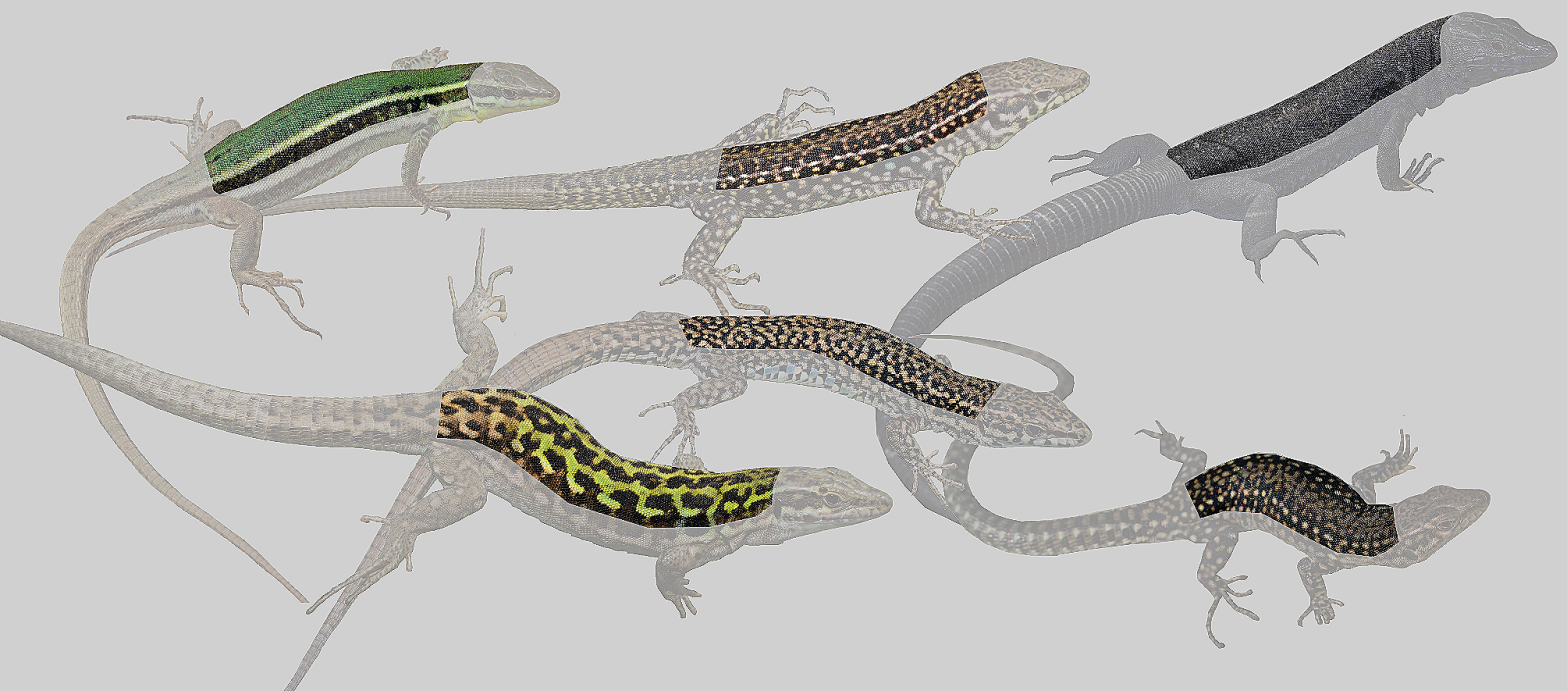
Examples of the variability of dorsal colouration in the genus *Podarcis*. Top line, island endemics, right to left: *Podarcis waglerianus* (Sicily), *Podarcis tiliguerta* (Sardinia), and *Podarcis lilfordi* (Illa de l’Aire, Menorca). Bottom line, mainland species, right to left: *Podarcis siculus* (Italian Peninsula), *Podarcis liolepis* (Iberian Peninsula), and *Podarcis lusitanicus* (Iberian Peninsula). Photo credits: Daniel Escoriza

**Fig. 4:**
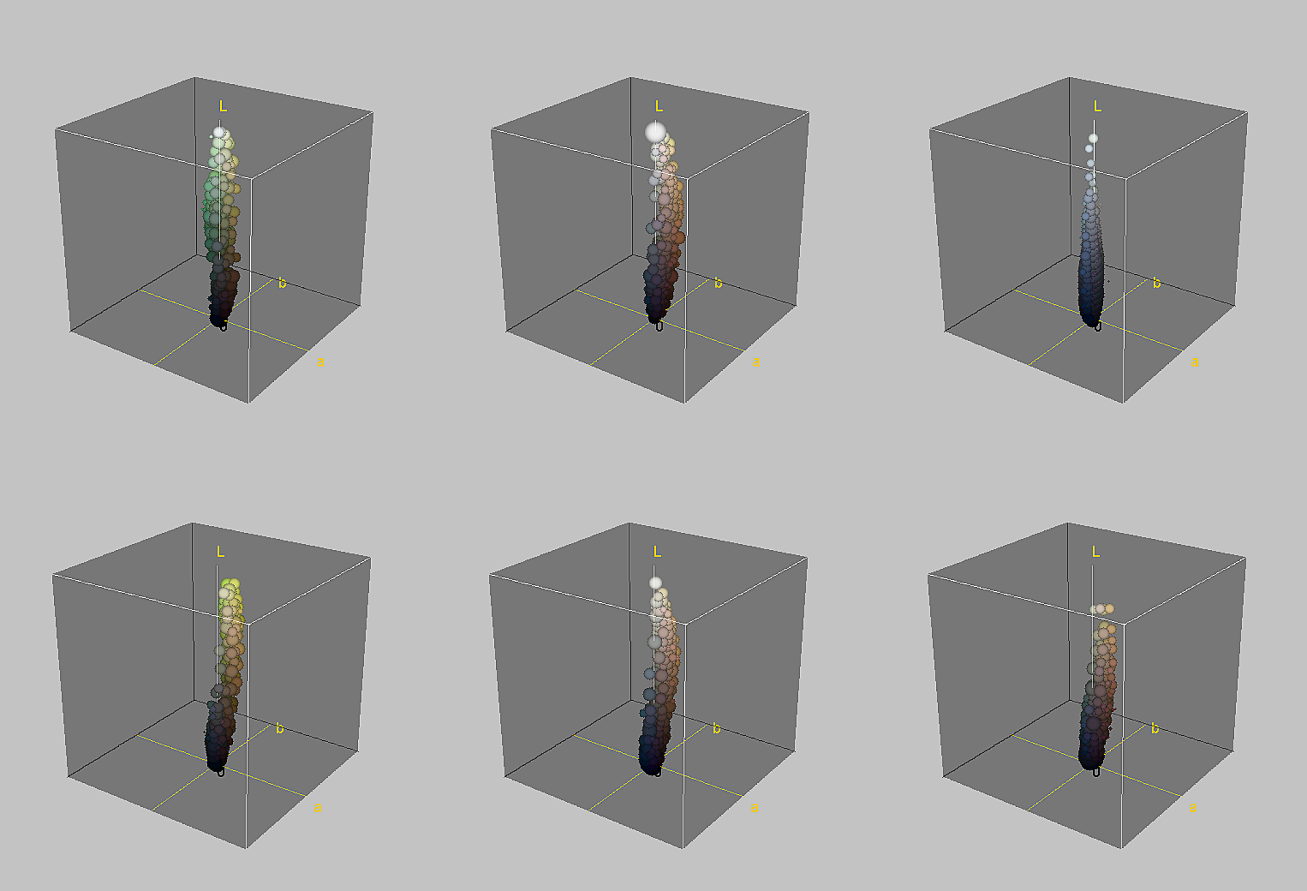
Examples of dorsal colour decomposition in CIELAB space. Top line, island endemics, right to left: *Podarcis waglerianus* (Sicily), *Podarcis tiliguerta* (Sardinia), and *Podarcis lilfordi* (Illa de l’Aire, Menorca). Bottom line, mainland species, right to left: *Podarcis siculus* (Italian Peninsula), *Podarcis liolepis* (Iberian Peninsula), and *Podarcis lusitanicus* (Iberian Peninsula)

Colouration variations were evaluated using photographs taken by the author (5%) and images provided by a web-based community (95%) (Additional File 1). These images were taken from iNaturalist [45], which contains thousands of georeferenced photographs with their level of spatial error. These data are reliable for studies on the composition of faunal assemblages and species colouration [46–48]. However, given the heterogeneity in the quality of the images, those that did not meet the following minimum requirements were discarded: (1) the quality of the image had to enable verification of taxonomic identities; (2) the colour image could not show extremely low or high light; (3) the colour image could not be blurry or grossly pixelated (e.g., the small dorsal scales had to be distinguished); (4) the colour image was georeferenced with a maximum error of 1000 m; and (5) the image corresponded to live, not preserved, animals. The author’s photographs included in the study were taken with a Nikon D3400 reflex camera with a Nikkor 18–55 mm lens and an SB-700 speedlight flash. In total, 1400 images of 27 species of lacertids were used (Figs. 2, 3 and 4). Color fidelity between author’s images and the database was assessed using PERMANOVA test on a stratified random sample (*n* = 100, 50% each source) of CIELAB coordinates for a single species. No significant difference was found in 70% of resamplings. To evaluate the phylogenetic effect on colour variation, we built a consensus phylogenetic tree using published phylogenies [49], using iTol [50]. The phylogenetic tree is shown in Additional File 2.

**Fig. 5:**
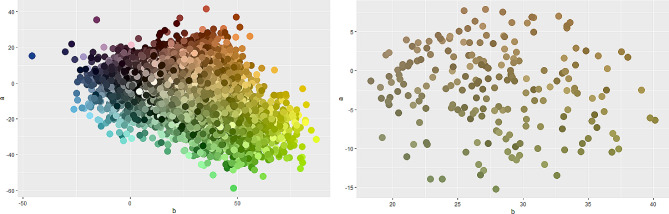
Colour decomposition in CIELAB space for 1400 individuals and 27 species of the genus *Podarcis*. Variation is shown for the a-axis (green–red) and b-axis (blue–yellow). Right, full range of 14,000 colours; left, 200 nearest colours around the two centroids for the genus *Podarcis*

### Colour data and variables

The method proposed by Laitly et al. [51] was utilised to define the colours that make up each image. Ten random points within a polygon encompassing the region of interest (ROI) were generated using ImageJ [52]. Laitly et al. [51] proposed using a minimum of three points to compare the plumage colouration of birds, but in this study, I used a larger number given the complexity of the dorsal pattern in lizards. At each of these points, the coordinates for the CIELAB colour scale were obtained [51]. ROI was defined as the rectangular polygon limited longitudinally between the margin of the cephalic scales and the base of the hind legs and laterally by the costal sides of the body (Fig. 2). In this genus of lacertids, dimorphic sexually reproductive colouration manifests primarily in the throat and ventral regions, with dorsal colouration showing weak dependence on reproductive state [36]. The CIELAB scale decomposes colours in a tri-dimensional space: on the *l*-axis (lightness), on the *a*-axis (green-red) and on the *b*-axis (blue-yellow) [53]. The CIELAB axes were used as response variables in the regression analyses.

Two additional variables were also generated: individual similarity to the genus averages (or colour centroids) and individual colour contrast. The former variable explores pattern variations on islands compared to their mainland counterparts. The similarity to the genus colour centroids quantified the individual distance to the 200 colours closest to the two genus colour centroids (Fig. 5). This distance was calculated using Gower’s similarity [54]. The two centroids were selected after visualisation confirmed that two basic colours (green and brown) were predominant within the 14,000-colour range (Fig. 4). These analyses were performed using robust K-means clustering [55] in R [56]. Individual colour contrast was assessed by comparing the similarity among the 10 colours obtained at the individual level using the CIEDE2000 formula [57]. In this method, an individual of uniform colour scores lower than one with a disruptive pattern (dark speckled or stripped). This variable was estimated using the farver package [58] in R.

### Data analyses

The analyses (i) quantified the amount of phylogenetic signal in chromatic variations and (ii) tested their statistical association with environmental predictors. The statistical significance of the phylogenetic signal was determined using Pagel’s λ [59]. Pagel’s λ quantifies the level to which the expression of a character is constrained by phylogenetic relationships (highly constrained, λ approach 1) or not (lowly constrained, λ approach 0) [59]. The value of λ was calculated after 10,000 resamplings of the response matrices [60]. This analysis was performed using the phytools package [61] in R.

The associations between environmental properties and chromatic variation were visualised using principal component analysis (PCA) after the logarithmic transformation of the variables [62]. The statistical significance of these associations was tested using generalised least squares (GLS) regression. GLS enables the incorporation of spatial information into regression models using correlation structures and nugget effects [63]. The models were built with different spatial correlation parameters (exponential, Gaussian, linear, spherical, and rational quadratics) that removed the effect of spatial interdependence of the data [64]. The best model (including one without spatial correlation parameters) was selected using Akaike’s iterative procedures (Burnham and Anderson, 2002) [65]. These analyses were performed with nlme (Pinheiro et al., 2020) [66] in R. Bonferroni-adjusted alpha levels were used for regression tests.

### Electronic supplementary material

Below is the link to the electronic supplementary material.

Supplementary Material 1

Supplementary Material 2

## Data Availability

Data will be made available on request.
