# Supplementary figures and images for: Environmental colour pattern variation in Mediterranean Podarcis
Source: BMC Ecol Evol. 2024 Apr 25;24:53. doi: 10.1186/s12862-024-02242-1 (PMC11044340; doi:10.1186/s12862-024-02242-1)

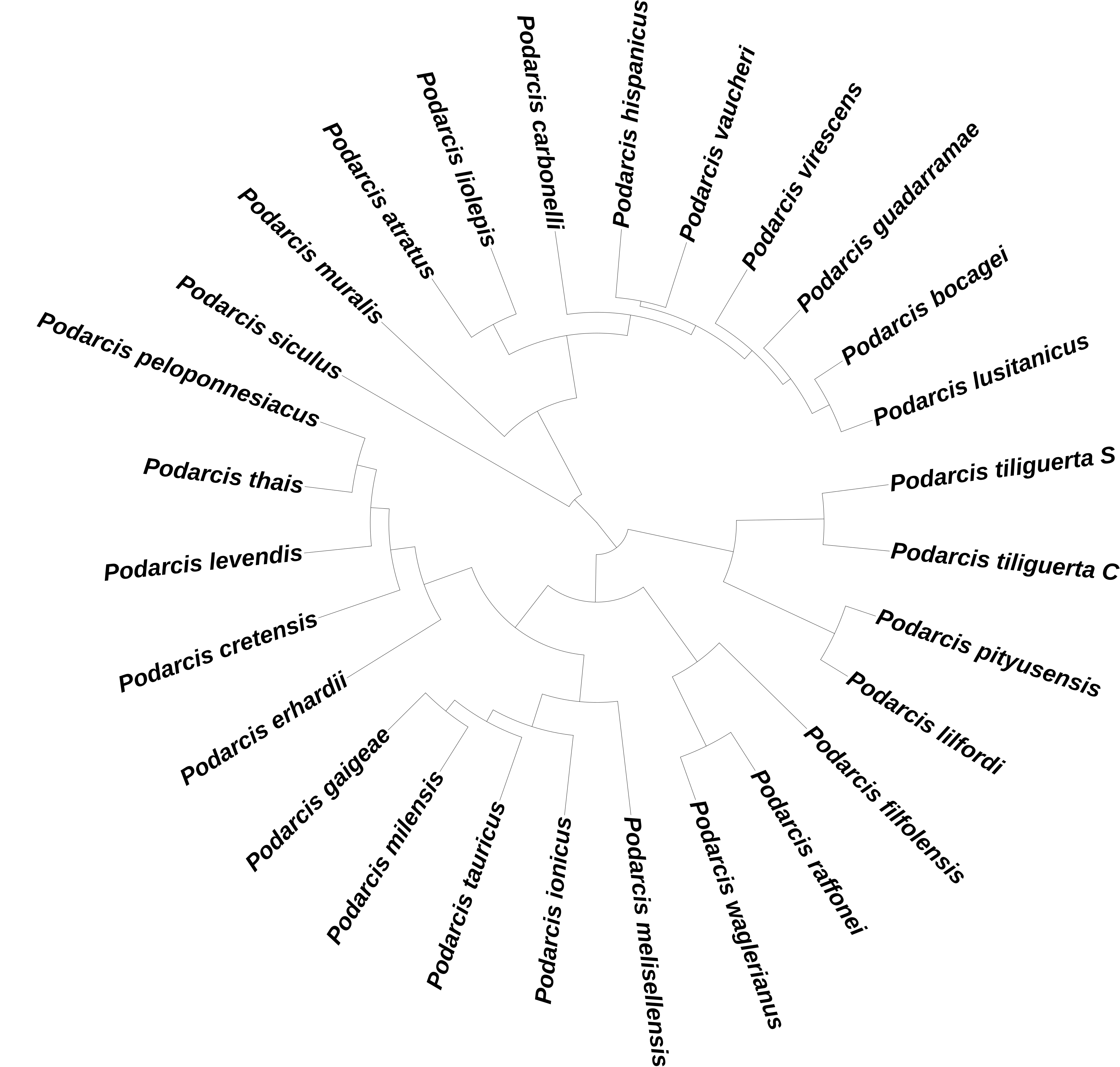

Supplement: Supplementary file 2 — Supplementary Material 2 [file 12862_2024_2242_MOESM2_ESM.png]
